# Supplementary material for: Characterization of the Duodenal Mucosal Microbiome in Obese Adult Subjects by 16S rRNA Sequencing
Source: Microorganisms. 2020 Mar 29;8(4):485. doi: 10.3390/microorganisms8040485 (PMC7232320; doi:10.3390/microorganisms8040485)
Supplement: Supplementary file 1 [file microorganisms-08-00485-s001.zip › microorganisms-730546 suppl final verison 0329/microorganisms-730546 suppl final.pdf]

*microorganisms*

MDPI

**Supplemental Materials and Methods**

*Sampling of duodenal biopsies by endoscopy:*

In this step including an appropriate negative control is no easy task, particularly when the studied samples are faeces or biopsies. In fact, sampling the air or container with a different instrument could not be a true negative control since no material should be available for DNA extraction. Consequently, for this step we did not have negative controls, but our goal was to minimize technical variation as below reported.

In order to reduce contamination risk all biopsies were performed using standardized procedures. In particular, all endoscopes were reprocessed before use according to high-level disinfection protocols including manual washing, automated endoscope washer reprocessing and adequate drying/storage after rinsing (Multisociety guideline on reprocessing flexible gastrointestinal endoscopes [53]) defined as the destruction of all vegetative microorganisms, mycobacteria, small or nonlipid viruses, medium or lipid viruses, fungal spores and some, but not all, bacterial spores. All samples were taken with sterile biopsy forceps and the endoscopist wore sterile gloves and a mask. The samples used for the study were invariably taken before the other samples used for histological examination. Oral washing was performed for all patients before endoscopic study of enrolled patients: oral contamination can be considered the smallest possible, although unavoidable. After endoscopic biopsy, specimen was carefully extracted, without oral or gastroesophageal passage or contact. All single-use containers were used for all biopsies. All specimens were immediately placed in a sterile tube on dry ice and transferred to a –80°C freezer within 15 min and until DNA extraction. The same procedure was performed in the two different clinical centers which contributed equally to the enrolment of both obese and controls groups. We retain that this sampling procedure was effective in minimizing risk of contamination, either external either internal.

*Sample processing*

All our samples (both from control and obese subjects) were processed concurrently in the same laboratory at Center of advanced Biotechnology CEINGE scarl, center that has implemented and maintains a quality management system which complies with the standard UNI EN ISO 9001:2015 for the diagnostic activities reported below, among which Genomix (next generation sequencing).

(See attach below for reviewer's use).

In this framework, the same researcher, reagents, and equipment were used to process all the samples of the present study. Personnel wore protective clothing and equipment to cover all exposed surfaces (i.e., disposable gloves, lab-coat, face-mask) and processed samples in controlled environment, as recently suggested [51].

*DNA extraction*

The DNA extraction of all samples was performed in a pre-PCR designed room under a laminar-flow hood to protect laboratory staff, samples and experiments from contaminations. Pre-PCR wok was physically isolated from the post-PCR work. We extracted DNA from all samples by using the same QIAamp DNA mini kit and according manufacter's instructions. The same kit was previously used by us and other groups for duodenal microbiome studies in another disease model [54,55]. DNA quantity was evaluated with the NanoDrop® ND-1000 UV-Vis spectrophotometer (NanoDrop Technologies, Wilmington, DE, USA). All DNA were diluted at 50 ng/μl for following PCR.

*Primer choice*

In this study, we used primers able to amplify the V4-V6 regions of the 16S rRNA. These primers were chosen after in-deep study of the literature. In particular: (i) the V4 region of 16S rRNA gene has been highly recommended as the gold standard for profiling of human gut microbiome by the MetaHIT consortium [10]; (ii) the V4-V5 hypervariable regions have shown to achieve a more accurate bacterial identification [56] and (iii) the V4-V6 region has shown the best performance for gut microbiome profiling considering also the size of the amplicon [57]. In addition, the same primers have been already used by our group for other metagenomic studies [39,55,58]. Finally, the primers were tested on primer blast (https://www.ncbi.nlm.nih.gov/tools/primer-blast/), using as reference the human genome, and no match were found, indicating no aspecific amplifications. Probe match analysis to verify taxa specificity was also carried out using both RDP database (https://rdp.cme.msu.edu), that found 1,188,968 hits within the domain "Bacteria", and Silva TestPrime, that found 71% of match on Bacteria.

*PCR 16S rRNA*

The PCR was carried out in a PCR designed room under a PCR hood with HEPA-filtered vertical laminar flow to ensure the contamination control and PCR process repeatability. An integrated UV lamp enabled rapid decontamination of the work zone between experiments and prevents cross-contamination. Sterile water (Molecular Biology Grade Water, Corning) was used for reagent mix preparation and as negative control. During PCR reaction for 16S rRNA the negative control was amplified and no amplification was obtained. During PCR reaction for 16S rRNA a negative control was amplified and no amplification was obtained on a 2% agarose gel indicating the absence of contamination.

*PCR Purification and Quantitation*

The AMPure XP beads were used to purify the 16S V4 and V6 amplicon from contaminants (dNTPs, salts, primers, primer dimers).The quality assessment of PCR products was performed by TapeStation (Agilent Technologies, Santa Clara, California, USA).

PCR product quantity was assessed by Qubit dsDNA BR assay (Thermo Fisher, Waltham, Massachusetts, USA) according to the manufacturer's instructions. The average of 2 measurements was used to dilute the samples at 0,2 ng/µl for the next step.

*Index PCR*

This step attaches dual indices and Illumina sequencing adapters using the Nextera XT Index Kit. This step was carried out in a post-PCR designed room under a PCR hood. No negative or positive controls were used. A step of purification (with AMPure XP beads to clean up the final library before quantification) and the quality assessment on Tape station were performed.

*Library Quantification, Normalization, and Pooling*

Each amplicon of previous step was quantified by Qubit dsDNA HS assay (Thermo Fisher, Waltham, Massachusetts, USA) according to the manufacturer's instructions. The average of 2 measurements was used to pool all the amplicons in equimolar amount.The concentration of the pool was 4 nM; this pool was diluted to 8 pM for sequencing.

*Data processing*

The strategy of using only the forward reads in case of scarcely overlapping paired ends is a very common option, as well as the concatenation that we chose to not use for the reasons explained in the methods section. It is also suggested by the software developer (as example please see https://www.drive5.com/usearch/manual/merge_badrev.html). Software allowing the use of non overlapping reads, as IM-TORNADO [59], treat the forward and reverse reads separately and pair what is possible to pair. Then the pipeline goes in parallel for the three different input files (R1,R2 and paired) and provide three different results. We think that since the R1 and R2 reads map to two

different regions is not recommendable to use them separately. Furthermore the V4 region is much more informative than the V6 [60], as also demonstrated by the fact that the results are confirmed when the R2 reads are added, as in the case of the alternative strategy we tested and cited in the methods, joining paired (obtained by qiime join_paired_ends.py), R1 and R2 reads. Here the intersection of the significative genera obtained adding the reverse reads (join+R1+R2) and using only the forward reads (R1). Only one more significative genus is obtained: Megasphaera (p: 0.046), but given the lack of consensus we decided to not consider it.

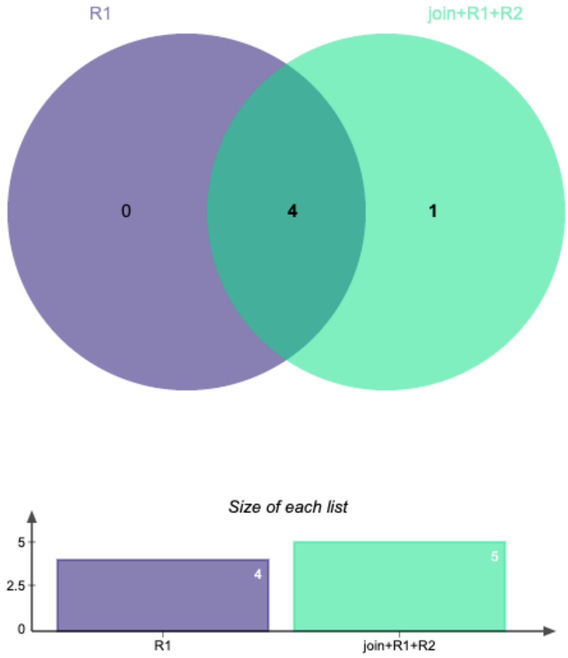


Common elements in DADA2_R1 and join+R1+R2 : Oribacterium; Atopobium; Stomatobaculum; Bifidobacterium

**Table 1.** Sequences and taxa information relative to the 16S sequencing data processing.

SEQUENCES

| | | input | filtered | denoised | nonchim |
|---|---|---|---|---|---|
| Mean | **CO** | 144346.88 | 39367.19 | 39023.56 | 37379.13 |
| | **OB-1** | 124758.00 | 31505.92 | 31185.23 | 30319.77 |
| | **OB-2** | 120679.17 | 33314.83 | 32970.83 | 31968.17 |
| | | | | | |
| SEM | **CO** | 12786.23 | 8434.69 | 8396.77 | 7384.48 |
| | **OB-1** | 10252.05 | 2436.66 | 2422.07 | 2177.98 |
| | **OB-2** | 11862.02 | 3962.57 | 3980.36 | 3776.13 |

TAXA

| | | Phylum | Class | Order | Family | Genus | Species |
|---|---|---|---|---|---|---|---|
| Total | **CO** | 18 | 32 | 63 | 105 | 212 | 134 |
| | **OB-1** | 15 | 29 | 56 | 97 | 188 | 111 |
| | **OB-2** | 14 | 28 | 52 | 95 | 143 | 77 |
| | | | | | | | |
| Mean > 1% | **CO** | 4 | 9 | 12 | 14 | 15 | 6 |
| | **OB-1** | 6 | 10 | 14 | 19 | 18 | 3 |
| | **OB-2** | 6 | 10 | 14 | 16 | 17 | 6 |

Regarding sequences, the average amount and the related SEM of input (raw), quality filtered, denoised by Dada inference algorithm, and chimeras free reads are shown. Regarding taxa, for each of the three groups the amount of the 7 taxonomic levels are reported, both total and with only taxa having an abundance >1%.
